# Utility of a pediatric observation unit for the management of children admitted to the emergency department

**DOI:** 10.1186/s13052-021-00959-z

**Published:** 2021-01-18

**Authors:** Antonio Gatto, Serena Rivetti, Lavinia Capossela, Davide Pata, Marcello Covino, Antonio Chiaretti

**Affiliations:** 1 Institute of Pediatrics, Fondazione Policlinico Universitario A. Gemelli IRCCS, Largo A. Gemelli, 00168 Rome, Italy; 2 Institute of Pediatrics, Fondazione Policlinico A. Gemelli IRCCS - Università Cattolica Sacro Cuore, Rome, Italy; 3 Department of Emergency, Fondazione Policlinico Universitario A. Gemelli IRCCS - Università Cattolica Sacro Cuore, Rome, Italy

**Keywords:** Pediatric observation unit, Children, Emergency department

## Abstract

**Background:**

Observation Units (OU), as part of emergency department (ED), are areas reserved for short-term treatment or observation of patients with selected diagnoses to determine the need for hospitalization or home referral.

**Methods:**

In this retrospective cohort study, we analyzed similarities and differences of children admitted to the pediatric ED of the Fondazione Policlinico Universitario A. Gemelli IRCCS hospital in the first 2 years of OU activity, analyzing general patient characteristics, access modalities, diagnosis, triage, laboratory and instrumental examinations, specialist visits, outcome of OU admission and average time spent in OU. Furthermore, we compared total numbers and type of hospitalization of the first 2 years of OU activity with those of previous 2 years.

**Results:**

The most frequent diagnoses were abdominal pain, minor head injury without loss of consciousness, vomiting, epilepsy and acute bronchiolitis. The most performed laboratory examinations were blood count. The most commonly performed instrumental examination was abdominal ultrasound. Neurological counseling was the most commonly requested.

Average time spent in OU was 13 h in 2016 and 14.1 h in 2017. Most OU admissions did not last longer than 24 h (90.5% in 2016 and 89.5% in 2017).

In the years 2014–2015, 13.4% of pediatric patients accessing the ED were hospitalized, versus 9.9% the years 2016–2017 reducing pediatric hospital admissions by 3.6% (*p* <  0.001).

**Conclusions:**

This study demonstrate that OU is a valid alternative to ordinary wards for specific pathologies. In accordance with the literature, our study showed that, in the first 2 years of the OU activity, admissions to hospital ward decreased compared with the previous 2 years with an increase of complex patients.

## Background

Observation units (OU) are dedicated clinical areas to observe or temporary treat patients admitted to the emergency department (ED) with selected diagnoses, providing an alternative treatment site [1] to determine the need for hospitalization or home referral.

OUs have their origins in the field of observational medicine and exist since the sixties of the last century to respond to organizational and economic needs. Since then, OUs are well established in adult medicine. For example, the introduction of a short observation period improved the diagnosis of myocardial infarction, reducing the number of unidentified heart attacks in adults with chest pain [1]. As far as atrial fibrillation and tachycardia are concerned, a brief observation period increased safety for patients about discharge from the ED [2, 3].

Instead, pediatric OU were first introduced in the United States, given the benefits achieved in adult medicine. Pediatric OUs allow efficient protocol-based observation and treatment of children who arrive at the pediatric emergency room for approximately 24–36 h, ensuring high intensity and quality of care.

Several pediatric diseases require observation or hospitalization of few hours and about one third of admissions in pediatric wards are short-term with “observation status” [3, 4]. According to some authors, patients with asthma, croup, dehydration and convulsions benefit greatly from a short observation period at the pediatric OU; at the same time hospitalization can be avoided and high quality of care maintained [5]. Interestingly, Greenberg et al. assessed the use of Pediatric Intensive Brief Observation as an alternative to hospitalization in children with croups, highlighting a reduction in the number of hospitalizations [6].

Miescier et al. highlighted that many children with asthma treated in an OU were discharged within 24 h and only 25% of them needed to hospitalization to continue treatment [7].

Plumb et al. identified the pediatric OU as a valid alternative to hospitalization or intensive care for patients from the ED who ingested toxicants allowing a 24-h observation before discharge [8].

Considering these observations, the Fondazione Policlinico Universitario Agostino Gemelli IRCCS hospital, a third level hospital located in Rome, implemented a pediatric OU in January 2016.

The aim of this study was to evaluate OU activity in terms of service provision and effects on hospitalization to pediatric wards in its first 2 years of activity.

## Methods

In this retrospective cohort study, data of all pediatric patients (0–17 years), admitted to the pediatric ED, between January 2014 to December 2015, 2 years prior to the pediatric OU activation, and between February 2016 to December 2017, during the first 2 years of OU activity, were analyzed based on information of their medical records.

We extracted these records from GIPSE database. GIPSE is the software used in ED. These records are anonymous and available for the medical staff of the Fondazione Policlinico Universitario Agostino Gemelli IRCCS hospital, using their personal account.

Data concerning the following elements were collected regarding patients admitted to the pediatric OU during 2016 and 2017:

1. Triage: based on the patient’s general condition, a color code was assigned to patients admitted to the ED, corresponding to urgency degree and priority level
  Red code: emergency, immediate life threatening, absolute priority;
  Yellow code: urgency, serious injury, maximum effort to reduce patient waiting time,
  Green code: minor urgency, apparently not life threatening, “deferrable” intervention,
  White code: no urgency, apparently not serious, visit carried out when possible, compatibly with all other emergencies.
2. Diagnosis/symptoms.
3. Laboratory examinations.
4. Instrumental examinations.
5. Specialist visits.
6. Intervention outcome and average time spent in OU.

Data of 2016 were compared with those of 2017 to highlight similarities and differences. Secondly, to evaluate the effect of OU activation on pediatric hospital admissions, rates and hospitalization types of patients admitted to the ED in the years 2014–2015 were compared with data of patients admitted to the ED in the years 2016–2017, when the pediatric ED had a new pediatric OU. For the admissions of patients to the pediatric ward, criteria from the international guidelines for each disease were used. Among patients admitted to the OU, the admission to the pediatric ward was considered if their status needed to an observation longer than 36 h or more examinations than those done in OU for diagnosis or if they needed to surgery or longer treatments.

Data concerning the triage color code assigned to hospitalized patients during the two period 2014–2015 and 2016–2017 were compared. Furthermore the main diagnoses of patients admitted to the pediatric ward from the ED in the 2014–2015 period were compared with the main diagnoses observed in hospitalized patients in the years 2016–2017.

### Statistical analysis

Data concerning categorical variables are expressed in absolute numbers and percentages. Continuous variables are expressed as mean ± standard deviation.

Comparison between groups of categorical variables was carried out using Chi-square test with Yates correction. A value of *p* <  0.05 was required for statistical significance.

## Results

Table 1 shows data related to ED admission of patients treated in the OU between February 2016 to December 2017; 27,351 patients were evaluated; 1610 (5.8%) patients were treated in the OU, 746 (46.3%) were females and 864 (53.7%) were males with an average age of 6.9 (±5.8) years.

**Table 1:** Access modality, priority to triage and diagnosis of patients admitted to OBI

	2016	2017	TOTAL
**Number of patients admitted in OU**	**859**	**751**	**1610**
**Priority to triage**	**n (%)**	**n (%)**	**n (%)**
Green code	456 (53.1%)	341 (45.4%)	797 (49.5%)
Yellow code	380 (44.2%)	348 (46.3%)	728 (45.2%)
Red code	19 (2.2%)	58 (7.7%)	77 (4.8%)
Not done	4 (0.5%)	0	4 (0.2%)
White code	0 (0%)	4 (0.5%)	4 (0.2%)
**Diagnosis**	**n (%)**	**n (%)**	**n (%)**
Abdominal pain	91 (10,6%)	70 (9,3%)	161 (10%)
Minor head injury without loss of consciousness	67 (7,8%)	56 (7,5%)	123 (7,6%)
Vomit/Dehydration	79 (9.2%)	49 (6,5%)	128 (7.9%)
Epilepsy	45 (5,2%)	45 (6%)	90 (5,6%)
Acute bronchiolitis	43 (5%)	42 (5,6%)	85 (5,3%)
Febrile convulsion	30 (3,5%)	21 (2,8%)	51(3,2%)
Convulsions	29 (3,4%)	24 (3,2%)	53 (3,3%)
Temperature	29 (3,4%)	26 (3,5%)	55 (3,4%)
Diarrhea	26 (3%)	17 (2,3%)	43 (2,7%)
Moderate head injury (GCS 8–13)	26 (3%)	25 (3,3%)	51 (3,2%)
Acute appendicitis	23 (2,7%)	17 (2,3%)	40 (2,5%)
Ingestion of chemicals	23 (2,7%)	29 (3,9%)	52 (3,2%)
Extrinsic asthma with exacerbation	16 (1,9%)	11 (1,5%)	27 (1,7%)
Headache	15 (1,7%)	10 (1,3%)	25 (1,5%)
Bacterial pneumonia	13 (1,5%)	12 (1,6%)	25 (1,5%)
Syncope and presyncope	13 (1,5%)	12 (1,6%)	25 (1,5%)
Other diagnoses	291 (33,9%)	285 (37,8%)	576 (35,8%)

Regarding triage colour code, data from 2016 showed that 456 (53.1%) had a green code, 380 (44.2%) a yellow code and 19 (2.2%) a red color code. Data from 2017 showed that 341 (45.4%) patients had a green code, 348 (46.3%) a yellow code and 58 (7.7%) a red code.

Regarding diagnoses and symptoms at time of ED admission of patients treated in the pediatric OU, the most common diagnoses were abdominal pain in a non-specific location, minor head injury without loss of consciousness, vomiting, epilepsy and acute bronchiolitis.

We observed a difference between males and females regarding the most common diagnoses. For females, the most common diagnoses/symptoms were abdominal pain, 52 (12.7%) patients in 2016 and 41 (12.2%) patients in 2017, vomiting/dehydration, with 41 (10%) patients in 2016 and 24 (7.1%) in 2017, and minor head injury without loss of consciousness, with 23 (5.6%) patients in 2016 and 21 (6.3%) in 2017. In males, minor head injury without loss of consciousness was the most observed diagnosis/symptom with 44 (9.8%) patients in 2016 and 35 (8.4%) in 2017, the second one was abdominal pain, with 39 (8.7%) patients in 2016 and 29 (7%) in 2017, the third most common cause was vomiting/dehydration, with 38 (8.5%) patients in 2016 and 25 (6%) in 2017.

The most performed laboratory examinations in the first 2 years of activity of the pediatric OU remained unchanged. By far, the most frequent were blood count, performed in 597 (69.5%) patients in 2016 and 552 (73.5%) in 2017, blood chemistry tests, in 583 (67.9%) patients in 2016 and 546 (72.7%) in 2017, C-Reactive Protein, in 465 (54.1%) patients in 2016 and 376 (50.1%) in 2017, and blood coagulation tests, in 171 (19.9%) patients during 2016 and 235 (31.3%) in 2017 (Table 2).

**Table 2:** Evaluations and tests carried out for patients admitted to the OBI

	2016	2017	TOTAL
**Number of patients admitted in OU**	**859**	**751**	**1610**
**Laboratory tests**	**n (%)**	**n (%)**	**n (%)**
Complete blood count with differential white blood cell count	597 (69,5%)	552 (73,5%)	1149 (71,4%)
Blood chemistry tests (creatinine, total bilirubin, blood sugar, potassium, sodium, calcium, LDH, transaminase, amylase, creatine kinase)	583 (67,9%)	546 (72,7%)	1129 (70,1%)
CRP	465 (54,1%)	376 (50,1%)	841 (52,2%)
Blood coagulation tests (fibrinogen, PT and aPTT)	171 (19,9%)	235 (31,3%)	406 (25,2%)
Chemical urine test	120 (14%)	143 (19%)	263 (16,3%)
Troponin I ultra	70 (8,1%)	67 (8,9%)	137 (8,5%)
Blood group ABO, Rh	14 (1,6%)	24 (3,2%)	38 (2,4%)
βHCG	16 (1,9%)	21 (2,8%)	37 (2,3%)
Anti - EBV Ab	9 (1%)	21 (2,8%)	30 (1,9%)
Liquor chemical examination	16 (1,9%)	7 (0,9%)	23 (1,4%)
Blood culture	10 (1,2%)	8 (1,1%)	18 (1,1%)
Urine culture	10 (1,2%)	8 (1,1%)	18 (1,1%)
Toxicological urine (amphetamines, barbiturates, BDZ, cocaine, cannabinoids, methadone, opiates)	9 (1%)	16 (2,1%)	25 (1,6%)
HCG urine	9 (1%)	10 (1,3%)	19 (1,2%)
**Instrumental Tests**	**n (%)**	**n (%)**	**n (%)**
Abdominal ultrasound	192 (22,4%)	197 (26,2%)	389 (24,2%)
chest X-ray	145 (16,9%)	155 (20,6%)	300 (18,6%)
Brain CT	71 (8,3%)	94 (12,5%)	165 (10,2%)
Direct abdomen x-ray	32 (3,7%)	38 (5,1%)	70 (4,3%)
XR cervical spine	19 (2,2%)	23 (3,1%)	42 (2,6%)
XR lumbosacral column	14 (1,6%)	34 (4,5%)	48 (3%)
XR spine	13 (1,5%)	36 (4,8%)	49 (3%)
EEG	7 (0,8%)	14 (1,9%)	21 (1,3%)
XR pelvis	12 (1,4%)	26 (3,5%)	38 (2,4%)
CT cervical spine	10 (1,2%)	36 (4,8%)	46 (2,9%)
XR sacrococcygeal column	4 (0,5%)	16 (2,1%)	20 (1,2%)
**Specialistic examinations**	**n (%)**	**n (%)**	**n (%)**
Neurological consultation	102 (11,9%)	110 (14,6%)	212 (13,2%)
Otolaryngology consultancy	24 (2,8%)	27 (3,6%)	51 (3,2%)
Eye consultancy	14 (1,6%)	10 (1,3%)	24 (1,5%)
Resuscitation consultancy	9 (1%)	9 (1,2%)	18 (1,1%)
Poison control center consultancy	6 (0,7%)	5 (0,7%)	11 (0,7%)
Cardiology consultation	4 (0,5%)	6 (0,8%)	10 (0,6%)
Infectious disease consultancy	1 (0,1%)	2 (0,3%)	3 (0,2%)
Maxillofacial consultation	1 (0,1%)	10 (1,3%)	11 (0,7%)
Other consulations (urological, dermatological, urological)	45 (5,2%)	36 (4,8%)	81 (5%)

The most used instrumental examination was abdominal ultrasound, performed in 192 (22.4%) patients in 2016 and 197 (26.2%) in 2017; the second one was chest x-ray, done in 145 (16.9%) patients in 2016 and 155 (20.6%) in 2017. Finally, we found that brain CT was performed in 71 (8.3%) patients in 2016 and 94 (12.5%) in 2017 and direct abdomen X-ray in 32 (3.7%) patients in 2016 and 38 (5.1%) in 2017 (Table 2).

Regarding specialist consultations required for OU patients, neurological counseling was the most commonly requested, in 102 (11.9%) patients in 2016 and in 110 (14.6%) in 2017, followed by otolaryngology consultancy, in 24 (2.8%) patients in 2016 and 27 (3.6%) in 2017, and ophthalmology consultancy, performed in 14 (1.6%) patients in 2016 and 10 (1.3%) in 2017 (Table 2).

Average time spent in OU was 13 h in 2016 and 14.1 h in 2017. Most OU admissions did not exceed 24 h in 778 (90.5%) patients in 2016 and 672 (89.5%) in 2017. Specifically, admissions lasted between 6 and 12 h in 249 (29%) patients in 2016 and 177 (23.6%) in 2017 and shorter than 6 h in 203 (23.6%) patients during 2016 and 172 (22.9%) during 2017.

Finally, concerning admissions lasting longer than 24 h, an increase in those longer than 36 h treated in the OU was noted (13, 1.5%, in 2016 vs 28, 3.7%, in 2017).

Concerning the outcome of admission to the OU, 560 (65.2%) patients were discharged and referred to home care in 2016 compared with 463 (61.6%) in 2017. Instead, 250 (29.1%) patients in 2016 and 221 (29.4%) patients in 2017 were admitted to pediatric wards. Five (0.6%) patients in 2016 and 4 (0.5%) in 2017 were transferred to other facilities; those sent back to outpatient facilities were 15 (1.8%) in 2016 and 35 (4.7%) in 2017.

In this study, admission rates of pediatric patients admitted to the ED in the two-year period 2014–2015, the last 2 years of ED activity in the absence of the pediatric OU, was compared to admission rates from the pediatric ED to ward in 2016–2017, the first 2 years of the pediatric OU activity.

In the years 2014–2015, 21,225 pediatric patients were evaluated in the pediatric ED and 2867 (13.4%) were hospitalized, while in the years 2016–2017 a number of 27,351 patients was evaluated and 2710 (9.9%) of them were hospitalized. A reduction of 3.6% (*p* <  0.001) of the inpatient admission to ward was observed.

Among patients admitted to a pediatric ward from the ED during the 2 years period analyzed, this study showed almost the same frequency of yellow color code, 1005 (35,1%) in the years 2014–15 and 993 (36,6%) in the years 2016–17, but an increase of 5,3% of red color code, 95 (3,3%) in the years 2014–15 and 234 (8,6%) in the years 2016–17; it showed also a decrease of 6,2% for patients with green color code, 1349 (47,1%) in the years 2014–15 and 1109 (40,9%) in the years 2016–17 (Table 3).

**Table 3:** Patients admitted to ward in the period 2014–2015 compared to period 2016–2017. Value are expressed as absolute number (%) and compared by Chi2 test

	2014–2015	2016–2017	
**Number of patients admitted to ward**	**2867**	**2710**	
**Triage colour code**	**n (%)**	**n (%)**	***P*** **value**
Red code	95 (3,3%)	234 (8,6%)	< 0.001
Yellow code	1005 (35,1%)	993 (36,6%)	
Green code	1349 (47,1%)	1109 (40,9%)	
White code	0	16 (0,6%)	
Not done	418 (14,5%)	358 (13,2%)	

Main diagnoses of patients admitted to a pediatric ward from ED in the period 2014–2015 were compared with main diagnoses of the years 2016–2017 (Table 4). In particular, for the period 2016–2017 a reduction of 2,5% (*p* value < 0.001) of inpatient admissions for minor head injury was observed: 138 (4,8%) in the years 2014–15 and 61 (2,3%) in the years 2016–2017). A reduction of 2,3% (p value < 0.001) of inpatient admissions for abdominal pain was observed in the period 2016–2017: 136 (4,7%) in the years 2014–15 and 66 (2,4%) in the years 2016–2017. Furthermore a reduction of 2,4% (p value < 0.001) was observed in the number of patients admitted to a pediatric ward for vomiting in the period 2016–2017: 117 (4,1%) in the years 2014–15 and 45 (1,7%) in the years 2016–2017).

**Table 4:** Main diagnosis for admission to ward

Main diagnosis for admission to ward	2014–2015 n (%)	2016–2017 n (%)	***P*** value
Fever	207 (7,2%)	176 (6,5%)	0.32
Epilepsy	178 (6,2%)	166 (6,1%)	0.90
Minor head injury without loss of consciousness	138 (4,8%)	61 (2,3%)	**< 0.001**
Abdominal pain	136 (4,7%)	66 (2,4%)	**< 0.001**
Pneumonia	127 (4,4%)	129 (4,8%)	0.57
Vomiting	117 (4,1%)	45 (1,7%)	**< 0.001**
Convulsions	106 (3,7%)	79 (2,9%)	0.11
Acute bronchiolitis	104 (3,6%)	143 (5,3%)	**0.004**
Acute appendicitis	90 (3,1%)	147 (5,4%)	**< 0.001**
Febrile convulsions	81 (2,8%)	40 (1,5%)	**< 0.001**

## Discussion

The OU is an emerging care setting allowing intensive observation of patients accessing to the ED, who require more time to clarify their clinical condition or short-term treatment before discharge.

Amongst the advantages, OUs offer multidisciplinary patient management, with specialist consultations and diagnostic procedures in a short time, same as those reserved for ED patients, continuous (non-invasive) and close monitoring of vital parameters and treatment response, short-term observation, more direct relationship between patient and highly qualified medical and nursing staff.

Considering the main diagnosis evaluated in OU, Zebrach et al. identified acute gastroenteritis with dehydration (17% of observation patient admissions), occurring at twice the frequency of the next most common admission diagnoses, followed by orthopedic injury (9%) and asthma (8%) [9]. For Marks et al. [10] the main diagnosis was asthma (30%), followed by bronchiolitis (15%) and dehydration (13%), with only a small percentage of abdominal pain [11, 12]. In this study, the most frequent diagnoses were, in both years, non-specific abdominal pain, minor head injury without loss of consciousness, vomiting, epilepsy and acute bronchiolitis.

With regard to instrumental examinations, their frequency reflects the pathologies most frequently observed in OU.

The most commonly performed instrumental examination was abdominal ultrasound, consistent with the most frequent cause of admission to OU, which was abdominal pain. The second instrumental examination type was chest x-ray, probably associated with the fact that fever, bronchiolitis and bacterial pneumonia are common causes of admission to OU.

Finally, we found that the number of CT scans increased between 2016 and 2017, unlike what has been shown by other studies, in which the rate of CT use decreased by 30% as a result of OU institution, avoiding an unnecessary head CT for pediatric minor head injuries [13].

We have not observed any changes affecting the most frequently performed laboratory tests between the 2 years analyzed.

The median age and data for our study population are comparable to previously published reports on pediatric OUs [5–9].

According to other previous study, duration of the observation did not exceed 24 h, in the majority of cases, and most of the patients were discharged to their home after admission to the OU, underlining responsiveness of our OU to the specific clinical condition in a short time [5–15].

This study showed that during hospitalization in the OU, children received diagnostic and therapeutic intensive care, which also included the frequent request for specialist advice; therefore, our pediatric OU can be considered a “multidisciplinary advanced care unit”, where various pediatric specialties, from both clinical and surgical areas, evaluate patients. Generally, OUs can be configured as “intermediate unit of care”, an intermediate care setting between the hospital ward and the intensive care unit where patients receive close monitoring of vital signs and interventional medical-nursing assistance.

In a third level hospital, the structure and characteristics of OUs, could provide support for pathologies that need sub-intensive care and require higher levels of assistance than those provided in wards [16]. In this way, OUs can be seen as semi-intensive care units and alternatives to ward or in intensive care admission.

Using the pediatric OU, as a semi-intensive setting, would increase the availability of intensive care services for children urgently in need, which could result in fewer ward emergencies. We should also consider effects on the child’s quality of life because hospitalization in a pediatric OU is less aggressive with less stringent rules where the mother-child contact can be constantly maintained compared with intensive care admission.

From an economic point of view, in the absence of OUs, children with pathologies requiring a brief observation would be directly hospitalized in an ordinary ward, increasing the number of improper and short-term hospitalizations not remunerated by the national health system. Several studies reported a reduction of ordinary hospitalizations between 12 and 20.3% in the presence of an OU [9–17]. Our study revealed a 3.6% reduction of ordinary hospitalizations for patients admitted to the pediatric ED in the first 2 years of pediatric OU activity compared to the previous 2 years, which is in accordance with data of other studies. In particular, in a study conducted in an US pediatric hospital, a 2.5% decrease of hospitalizations was demonstrated after 2 years of pediatric OU activity [12].

We also noted an increase in the complexity of hospitalizations evidenced through reduced hospitalizations of patients with a green code and an increment of hospitalizations of yellow or red code patients. In particular, between 2014 and 2015, 21,225 children accessed to the pediatric ED and 13.5% (2867) of these were hospitalized in an ordinary hospital ward; amongst these 3.3% (95) patients had a red code, 35.1% (1005) a yellow code and 47.1% (1349) a green code.

In the two-year period 2016–2017, 27,351 patients accessed to the pediatric ED, 9.9% (2710) of these were admitted to the pediatric ward; 8.6% (234) of hospitalized patients received a triage red code, 36.6% (993) a yellow code and 40.9% (1109) a green code, which confirms the reduction of inappropriate hospitalizations.

In this study between 2014 and 2015 the main diagnosis for hospitalization was fever, followed by epilepsy, minor head injury without loss of consciousness and abdominal pain. Between 2016 and 2017 the main cause of hospitalization remained the fever followed by epilepsy, acute bronchiolitis and pneumonia. By comparing the main causes of inpatient admissions during the 2014–2015 and 2016–2017 periods, there was a reduction in admission rates to the pediatric ward for minor head injury, abdominal pain and vomiting in the latest 2 years with the introduction of OU.

Finally, in the absence of a pediatric Observation Unit, the patient would be hospitalized in ordinary wards to perform “clinical observation”. This leads to considerably higher costs and bed occupancy, which could be used for cases with higher indications of hospitalization [18–21].

## Conclusions

Our study demonstrated the usefulness of OUs as a valid alternative to ordinary ward hospitalization for selected pathologies. In accordance with literature, our study showed that, in the first 2 years of the OU activity, hospital ward admissions decreased compared with previous years, with an increased access of complex patients. This represents an important advantage regarding costs and resource allocation. In our experience, an increased ward bed availability was achieved, because admissions requiring short-term observation were reduced allowing bed allocation to patients in need of hospital care.

However, a series of critical issues emerged with the need: to establish precise hospitalization criteria and, subsequently, OU discharge criteria; to employ specialized staff providing high intensity pediatric care; to identify specific prognostic criteria to assess the quality of the pediatric OU activity.

Hence, further work needs to ensure and implement a service that appears to be at the forefront and that can have many positive implications to manage pediatric patient, both in terms of treatment effectiveness and efficiency in resource management.

## Data Availability

“Not applicable”.

## Abbreviations

OU: Observation Unit
ED: Emergency Department
CT: Computerized tomography
NHS: National Health Service
